# Supplementary material for: Effect of post COVID-19 on body composition, physical fitness, sleep quality and quality of life among young adults: a cross-sectional study of matched pairs
Source: PeerJ. 2024 Sep 20;12:e18074. doi: 10.7717/peerj.18074 (PMC11418813; doi:10.7717/peerj.18074)
Supplement: Supplemental Information 1 [file peerj-12-18074-s001.docx]

ลำดับที่................

**แบบคัดกรอง**

**กรุณาเติมข้อความลงในช่องว่างหรือทำเครื่องหมาย** ✓ **ในคำตอบที่คุณเลือก**

1. ท่านเป็นนักศึกษามหาวิทยาลัยวลัยลักษณ์ใช่หรือไม่

 ใช่

 ไม่ใช่

1. ท่านเคยได้รับเชื้อโควิด-19 และรักษาหายแล้วใช่หรือไม่

 ใช่

 ไม่ใช่

1. ท่านมีโรคประจำตัวหรือไม่

 ไม่มี

 ไม่เคยได้รับการตรวจ

 มี โปรดระบุ

 เบาหวาน  ไขมันในเลือดสูง

 ความดันโลหิตสูง  โรคหัวใจ

 โรคปอด  โรคภูมิแพ้

 อื่นๆ โปรดระบุ....................................................................

1. ตอบเฉพาะเพศหญิง ขณะนี้คุณมีภาวะเหล่านี้หรือไม่

 ตั้งครรภ์

 มีประจำเดือน

 ไม่มีภาวะใดๆเลย

1. โดยส่วนใหญ่หรือในช่วง 3 เดือนที่ผ่านมา ท่านมีพฤติกรรมการออกกำลังกายแบบใด

 ออกกำลังกายเบาๆ เช่น เดินเล่น ที่ทำให้หัวใจเต้นเร็วขึ้นและหายใจเร็วขึ้นเล็กน้อย

อย่างน้อย 60 นาทีต่อสัปดาห์

 ออกกำลังกายระดับปานกลาง เช่น เดินเร็ว ที่ทำให้หัวใจเต้นเร็วขึ้นและหายใจเร็วขึ้นปานกลาง

อย่างน้อย 150 นาทีต่อสัปดาห์

 ออกกำลังกายระดับหนัก เช่น วิ่ง เทนนิส ฟุตบอล ทำให้หัวใจเต้นเร็วขึ้นและหายใจเร็วขึ้นมาก

อย่างน้อย 75 นาทีต่อสัปดาห์

1. ท่านมีอาการอย่างไรบ้าง ระบุอาการทั้งขณะที่ติดเชื้อโควิด-19 และอาการในวันนี้ **(ทำเครื่องหมาย** ✓**ลงในช่องที่ตรงกับคำตอบของท่าน)**

| **อาการ** | **ขณะติดเชื้อโควิด-19** | **วันนี้** |
| --- | --- | --- |
| มีไข้ (อุณหภูมิสูงกว่า 37.5 องศา) |  |  |
| ไอ |  |  |
| เจ็บคอ/คอแห้ง |  |  |
| เจ็บหน้าอก |  |  |
| หายใจลำบาก/หายใจไม่อิ่ม/หายใจถี่ |  |  |
| ผมร่วง |  |  |
| เหนื่อยง่าย/อ่อนเพลีย/อ่อนล้า |  |  |
| การรับรสเปลี่ยน |  |  |
| จมูกไม่ได้กลิ่น |  |  |
| ปวดศีรษะ |  |  |
| คลื่นไส้/อาเจียน |  |  |
| ท้องเสีย |  |  |
| นอนหลับยาก/หลับไม่สนิท |  |  |
| ปวดเมื่อย |  |  |
| สับสน |  |  |
| หลงลืม/ความจำสั้น |  |  |
| อื่นๆ โปรดระบุ..................................... |  |  |
| อื่นๆ โปรดระบุ..................................... |  |  |

ลำดับที่................

**แบบสอบถาม**

**คำชี้แจง**

1. แบบสอบถามฉบับนี้จัดทำขึ้นเพื่อสำรวจปัจจัยต่างๆที่มีผลต่อการเข้ารับบริการทางการแพทย์ของผู้ที่มีอาการปวดหลังส่วนล่าง ประกอบด้วย 5 ส่วน ดังนี้

ส่วนที่ 1 : ข้อมูลพื้นฐาน

ส่วนที่ 2 : แบบสอบถามเรื่องสุขภาพ ฉบับภาษาไทยสาหรับใช้ในประเทศไทย (EQ-5D-5L)

ส่วนที่ 3 : ข้อมูลเกี่ยวกับอาการปวด

ส่วนที่ 4 : แบบประเมินคุณภาพการนอน (Pittsburgh sleep quality index score)

ด้วยเกียรติและจรรยาบรรณ ขอรับรองว่า จะรักษาข้อมูลของท่านไว้เป็นความลับ และจะประมวลผลในภาพรวมเท่านั้น

**กรุณาเติมคำตอบลงในช่องว่าง หรือ ทำเครื่องหมาย** ✓**ลงใน**  **หน้าข้อความที่ตรงกับคำตอบของท่าน**

**ส่วนที่ 1**: ข้อมูลพื้นฐาน

1. อายุ........................ปี..................เดือน
2. เพศ  ชาย  หญิง
3. สำนักวิชาที่เรียน...........................................................................
4. ปัจจุบันท่านออกกำลังกายหรือไม่

 ไม่ออกกำลังกาย

 ออกกำลังกาย โปรดระบุชนิดของการออกกำลังกาย

 เดิน  วิ่ง

 ฟุตบอล  ปั่นจักรยาน

 อื่นๆ (โปรดระบุ.........................................................)

1. คุณสูบบุหรี่หรือไม่

 ไม่สูบ (ข้ามไปตอบข้อ 7)

 เคยสูบ แต่เลิกแล้ว นาน (โปรดระบุ)........................ปี (ข้ามไปตอบข้อ 9)

 ปัจจุบันสูบ (โปรดระบุ)

 สูบทุกวัน โดยเฉลี่ยจำนวน.................................มวนวัน เป็นระยะเวลานาน.............ปี

 สูบบางวัน โดยเฉลี่ยจำนวน.................................มวนวัน เป็นระยะเวลานาน.............ปี

1. บุหรี่ที่คุณสูบเป็นชนิดใด

 ยาเส้น  ยาสูบ/ยาขาว

 อื่นๆ โปรดระบุ.....................................................................................

1. ท่านใกล้ชิด หรืออยู่อาศัยร่วมกัน หรือบ้านเดียวกัน กับผู้ที่สูบบุหรี่หรือไม่

 ใช่  ไม่ใช่

1. ช่วง 1 ปีที่ผ่านมา คุณนอนหลับพักผ่อนโดยเฉลี่ย............................ชั่วโมง/วัน
2. คุณคิดว่านอนหลับพักผ่อนในเวลากลางคืนเพียงพอหรือไม่

 เพียงพอ  ไม่เพียงพอ

1. ท่านทราบข้อมูลเกี่ยวกับโรคโควิด-19 หรือไม่ เช่น อาการ หรือ วิธีการปฏิบัติตนเพื่อป้องกัน

 ไม่ทราบ

 ทราบ

1. ท่านได้ปฏิบัติตนตามมาตรการการป้องกันโรคโควิด-19 อย่างเคร่งครัดหรือไม่ (ได้แก่ รักษาระยะห่าง ใส่แมส ล้างมือ วัดอุณหภูมิ และแสกนไทยชนะก่อนเข้า-ออกสถานที่สาธารณะทุกครั้ง)

 ไม่ปฏิบัติ

 ปฏิบัติบ้าง

 ปฏิบัติอย่างเคร่งครัด

1. ท่านได้รับวัคซีนป้องกันโรคโควิด-19 ครบถ้วนหรือไม่

 ครบ (โปรดระบุจำนวนเข็มที่ได้รับ......................................วันที่ฉีดเข็มล่าสุด.....................................)

 ไม่ครบ (โปรดระบุจำนวนเข็มที่ได้รับ......................................วันที่ฉีดเข็มล่าสุด...................................)

1. ท่านเคยต้องกักตัวเนื่องจากเป็นกลุ่มเสี่ยงสูงหรือไม่

 เคย

 ไม่เคย

1. ท่านเคยได้รับเชื้อโควิด-19 มาแล้วหรือไม่ กี่ครั้ง

 ไม่เคย

 1 ครั้ง ได้รับเชื้อเมื่อวันที่.......................................

กลุ่มผู้ป่วยสี  สีเขียว  สีเหลือง  สีแดง

การรักษาที่ได้รับ  รับประทานยารักษาตามอาการ

 รับประทานยา flavipiravir

 อื่นๆ โปรดระบุ.................................................................................

สถานที่ในการรักษาตัวขณะได้รับเชื้อ  บ้านพักของตนเอง

 ที่กักตัวในชุมชน หรือมหาวิทยาลัย

 โรงพยาบาลสนาม

 โรงพยาบาล

 2 ครั้ง ครั้งที่ 1 ได้รับเชื้อเมื่อวันที่.......................................

กลุ่มผู้ป่วยสี  สีเขียว  สีเหลือง  สีแดง

การรักษาที่ได้รับ  รับประทานยารักษาตามอาการ

 รับประทานยา flavipiravir

 อื่นๆ โปรดระบุ.................................................................................

สถานที่ในการรักษาตัวขณะได้รับเชื้อ  บ้านพักของตนเอง

 ที่กักตัวในชุมชน หรือมหาวิทยาลัย

 โรงพยาบาลสนาม

 โรงพยาบาล

ครั้งที่ 2 ได้รับเชื้อเมื่อวันที่.......................................

กลุ่มผู้ป่วยสี  สีเขียว  สีเหลือง  สีแดง

การรักษาที่ได้รับ  รับประทานยารักษาตามอาการ

 รับประทานยา flavipiravir

 อื่นๆ โปรดระบุ.................................................................................

สถานที่ในการรักษาตัวขณะได้รับเชื้อ  บ้านพักของตนเอง

 ที่กักตัวในชุมชน หรือมหาวิทยาลัย

 โรงพยาบาลสนาม

 โรงพยาบาล

 อื่นๆ โปรดระบุ.............................................................................................................

1. ท่านทราบสาเหตุของการติดเชื้อโควิด-19 หรือไม่

 ไม่ทราบ

 ทราบ โปรดระบุ.......................................................................................

**ส่วนที่ 2 แบบสอบถามเรื่องสุขภาพ ฉบับภาษาไทยสาหรับใช้ในประเทศไทย (EQ-5D-5L)**

ในแต่ละหัวข้อ กรุณาทำเครื่องหมาย ✓ ลงในช่องสี่เหลี่ยม (**)** เพียงช่องเดียว ที่ตรงกับสุขภาพของท่านใน**วันนี้** มากที่สุด

**การเคลื่อนไหว**

ข้าพเจ้าไม่มีปัญหาในการเดิน

ข้าพเจ้ามีปัญหาในการเดินเล็กน้อย

ข้าพเจ้ามีปัญหาในการเดินปานกลาง

ข้าพเจ้ามีปัญหาในการเดินอย่างมาก

ข้าพเจ้าเดินไม่ได้

**การดูแลตนเอง**

ข้าพเจ้าไม่มีปัญหาในการอาบน้ำ หรือใส่เสื้อผ้าด้วยตนเอง

ข้าพเจ้ามีปัญหาในการอาบน้ำ หรือใส่เสื้อผ้าด้วยตนเองเล็กน้อย

ข้าพเจ้ามีปัญหาในการอาบน้ำ หรือใส่เสื้อผ้าด้วยตนเองปานกลาง

ข้าพเจ้ามีปัญหาในการอาบน้ำ หรือใส่เสื้อผ้าด้วยตนเองอย่างมาก

ข้าพเจ้าอาบน้ำ หรือใส่เสื้อผ้าด้วยตนเองไม่ได้

**กิจกรรมที่ทำเป็นประจำ** (เช่น ทํางาน, เรียนหนังสือ, ทํางานบ้าน, กิจกรรมในครอบครัว หรือกิจกรรมยามว่าง) ข้าพเจ้าไม่มีปัญหาในการทำกิจกรรมที่ทำเป็นประจำ

ข้าพเจ้ามีปัญหาในการทำกิจกรรมที่ทำเป็นประจำเล็กน้อย

ข้าพเจ้ามีปัญหาในการทำกิจกรรมที่ทำเป็นประจำปานกลาง

ข้าพเจ้ามีปัญหาในการทำกิจกรรมที่ทำเป็นประจำอย่างมาก

ข้าพเจ้าทำกิจกรรมที่ทำเป็นประจำไม่ได้

**อาการเจ็บปวด/อาการไม่สบายตัว**

ข้าพเจ้าไม่มีอาการเจ็บปวดหรืออาการไม่สบายตัว

ข้าพเจ้ามีอาการเจ็บปวดหรืออาการไม่สบายตัวเล็กน้อย

ข้าพเจ้ามีอาการเจ็บปวดหรืออาการไม่สบายตัวปานกลาง

ข้าพเจ้ามีอาการเจ็บปวดหรืออาการไม่สบายตัวอย่างมาก

ข้าพเจ้ามีอาการเจ็บปวดหรืออาการไม่สบายตัวอย่างมากที่สุด

**ความวิตกกังวล/ความซึมเศร้า**

ข้าพเจ้าไม่รู้สึกวิตกกังวลหรือซึมเศร้า

ข้าพเจ้ารู้สึกวิตกกังวลหรือซึมเศร้าเล็กน้อย

ข้าพเจ้ารู้สึกวิตกกังวลหรือซึมเศร้าปานกลาง

ข้าพเจ้ารู้สึกวิตกกังวลหรือซึมเศร้าอย่างมาก

ข้าพเจ้ารู้สึกวิตกกังวลหรือซึมเศร้าอย่างมากที่สุด

เราอยากทราบว่าสุขภาพของท่านเป็นอย่างไรใน**วันนี้**

•  สเกลวัดสุขภาพนี้มีตัวเลขตั้งแต่ 0 ถึง 100


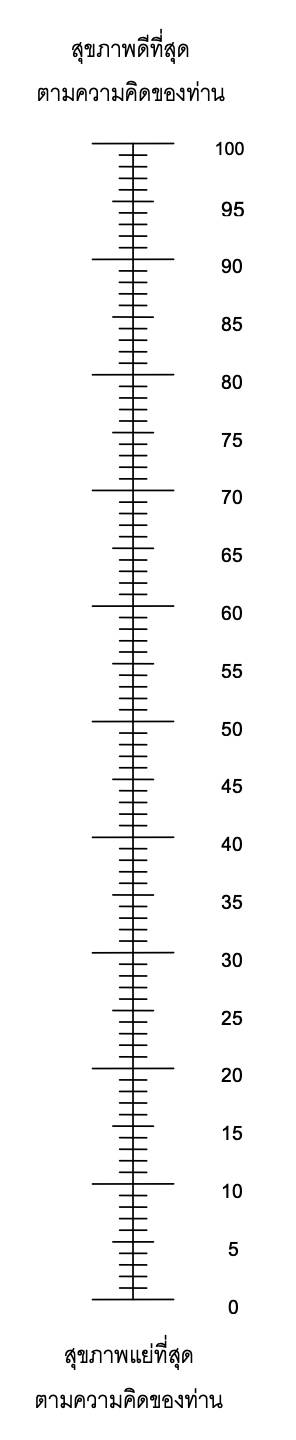
•  100 หมายถึง สุขภาพดีที่สุด ตามความคิดของท่าน

1. หมายถึง สุขภาพแย่ที่สุด ตามความคิดของท่าน

•  ทำเครื่องหมาย X บนสเกลเพื่อระบุว่าสุขภาพของท่านเป็นอย่างไรในวันนี้

•  ตอนนี้ กรุณาใส่ตัวเลขที่คุณได้ทำเครื่องหมายไว้บนสเกลในช่องสี่เหลี่ยมด้านล่างนี้

สุขภาพของท่านในวันนี้ =

**ส่วนที่ 3**: **ข้อมูลเกี่ยวกับอาการปวด**

1. โปรดระบุตำแหน่งที่มีอาการปวดในช่วง 12 เดือนที่ผ่านมาและในปัจจุบัน โดยทำเครื่องหมาย ✓ ในตารางที่ตรงกับคำตอบของท่าน (ระบุได้มากกว่า 1 ตำแหน่ง)

|  | | **12 เดือน**  **ที่ผ่านมา** | | **วันนี้** | |
| --- | --- | --- | --- | --- | --- |
|  |  | **มี** | **ไม่มี** | **มี** | **ไม่มี** |
| 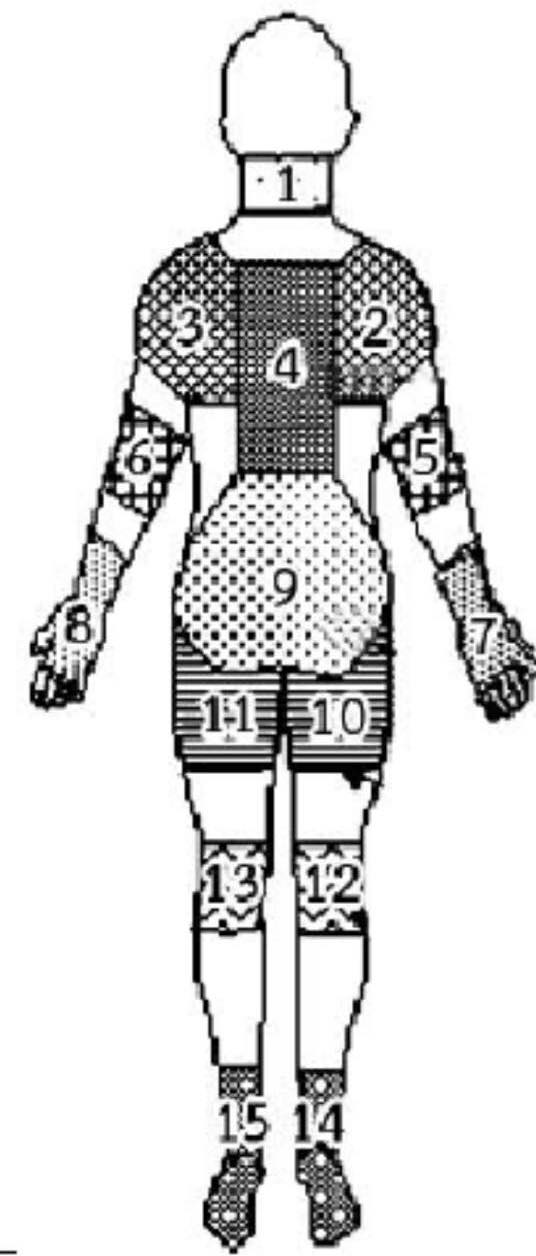 | 1. คอ |  |  |  |  |
|  | 1. ไหล่ขวา |  |  |  |  |
|  | 1. ไหล่ซ้าย |  |  |  |  |
|  | 1. หลังส่วนบน |  |  |  |  |
|  | 1. ข้อศอกขวา |  |  |  |  |
|  | 1. ข้อศอกซ้าย |  |  |  |  |
|  | 1. ข้อมือ/มือขวา |  |  |  |  |
|  | 1. ข้อมือ/มือซ้าย |  |  |  |  |
|  | 1. หลังส่วนล่าง |  |  |  |  |
|  | 1. สะโพก/ต้นขาขวา |  |  |  |  |
|  | 1. สะโพก/ต้นขาซ้าย |  |  |  |  |
|  | 1. เข่าขวา |  |  |  |  |
|  | 1. เข่าซ้าย |  |  |  |  |
|  | 1. ข้อเท้า/เท้าขวา |  |  |  |  |
|  | 1. ข้อเท้า/เท้าซ้าย |  |  |  |  |

**ส่วนที่ 4 แบบประเมินคุณภาพการนอน (Pittsburgh sleep quality index score)**

**คำแนะนำในการตอบแบบสอบถาม**

คําถามต่อไปนี้เกี่ยวข้องกับพฤติกรรมการนอนของท่านในช่วงระยะเวลา 1 เดือนที่ผ่านมา

คําตอบของท่านควรบ่งบอกสิ่งที่ใกล้เคียงความเป็นจริงมากที่สุด และเป็นสิ่งที่เกิดขึ้นกับตัว ท่านเป็นส่วนใหญ่ทั้ง ในเวลากลางวัน และกลางคืน โปรดตอบทุกคําถาม

1. ในช่วงระยะเวลา 1 เดือนที่ผ่านมา ส่วนใหญ่ท่านมักเข้านอนเวลากี่โมง

เวลาเข้านอน __________

1. ในช่วงระยะเวลา 1 เดือนที่ผ่านมาส่วนใหญ่ท่านต้องใช้เวลานานเท่าไร (นาที) จึงจะนอนหลับ

จำนวนนาที __________

1. ในช่วงระยะเวลา 1 เดือนที่ผ่านมา ส่วนใหญ่ท่านตื่นนอนตอนเช้าเวลากี่โมง

เวลาที่ตื่นนอนตอนเช้า __________

1. ในช่วงระยะเวลา 1 เดือนที่ผ่านมา ท่านนอนหลับได้จริงเป็นเวลากี่ชั่วโมงต่อคืน (คำตอบอาจแตกต่างจาก

ระยะเวลารวมทั้งหมดตั้งแต่เริ่มเข้านอนจนถึงตื่นนอน)

จำนวนชั่วโมงที่หลับได้จริงต่อคืน __________

โปรดตอบคำถามข้างล่างต่อไปนี้ทุกข้อ โดยแต่ละข้อให้เลือกตอบเพียง 1 คำตอบ

1. ในช่วงระยะเวลา 1 เดือนที่ผ่านมา
   1. นอนไม่หลับหลังจากเข้านอนไปแล้วนานกว่า 30 นาที

__________ ไม่เคยเลยในช่วงระยะเวลา 1 เดือนที่ผ่านมา

__________ น้อยกว่า 1 ครั้งต่อสัปดาห์

__________ 1 หรือ 2 ครั้งต่อสัปดาห์

__________ 3 ครั้งต่อสัปดาห์ขึ้นไป

- 1. รู้สึกตัวตื่นขึ้นระหว่างนอนหลับกลางดึกหรือตื่นเช้ากว่าเวลาที่ตั้งใจไว้

__________ ไม่เคยเลยในช่วงระยะเวลา 1 เดือนที่ผ่านมา

__________ น้อยกว่า 1 ครั้งต่อสัปดาห์

__________ 1 หรือ 2 ครั้งต่อสัปดาห์

__________ 3 ครั้งต่อสัปดาห์ขึ้นไป

- 1. ตื่นเพื่อไปเข้าห้องน้ำ

__________ ไม่เคยเลยในช่วงระยะเวลา 1 เดือนที่ผ่านมา

__________ น้อยกว่า 1 ครั้งต่อสัปดาห์

__________ 1 หรือ 2 ครั้งต่อสัปดาห์

__________ 3 ครั้งต่อสัปดาห์ขึ้นไป

- 1. หายใจไม่สะดวก

__________ ไม่เคยเลยในช่วงระยะเวลา 1 เดือนที่ผ่านมา

__________ น้อยกว่า 1 ครั้งต่อสัปดาห์

__________ 1 หรือ 2 ครั้งต่อสัปดาห์

__________ 3 ครั้งต่อสัปดาห์ขึ้นไป

- 1. ไอ หรือ กรนเสียงดัง

__________ ไม่เคยเลยในช่วงระยะเวลา 1 เดือนที่ผ่านมา

__________ น้อยกว่า 1 ครั้งต่อสัปดาห์

__________ 1 หรือ 2 ครั้งต่อสัปดาห์

__________ 3 ครั้งต่อสัปดาห์ขึ้นไป

- 1. รู้สึกหนาวเกินไป

__________ ไม่เคยเลยในช่วงระยะเวลา 1 เดือนที่ผ่านมา

__________ น้อยกว่า 1 ครั้งต่อสัปดาห์

__________ 1 หรือ 2 ครั้งต่อสัปดาห์

__________ 3 ครั้งต่อสัปดาห์ขึ้นไป

- 1. รู้สึกร้อนเกินไป

__________ ไม่เคยเลยในช่วงระยะเวลา 1 เดือนที่ผ่านมา

__________ น้อยกว่า 1 ครั้งต่อสัปดาห์

__________ 1 หรือ 2 ครั้งต่อสัปดาห์

__________ 3 ครั้งต่อสัปดาห์ขึ้นไป

- 1. ฝันร้าย

__________ ไม่เคยเลยในช่วงระยะเวลา 1 เดือนที่ผ่านมา

__________ น้อยกว่า 1 ครั้งต่อสัปดาห์

__________ 1 หรือ 2 ครั้งต่อสัปดาห์

__________ 3 ครั้งต่อสัปดาห์ขึ้นไป

- 1. รู้สึกปวด

__________ ไม่เคยเลยในช่วงระยะเวลา 1 เดือนที่ผ่านมา

__________ น้อยกว่า 1 ครั้งต่อสัปดาห์

__________ 1 หรือ 2 ครั้งต่อสัปดาห์

__________ 3 ครั้งต่อสัปดาห์ขึ้นไป

- 1. เหตุผลอื่นๆ ถ้ามี กรุณาระบุ

จากเหตุผลในข้อ 5.10 ในช่วงระยะเวลา 1 เดือนที่ผ่านมา เกิดบ่อยเพียงใด

__________ ไม่เคยเลยในช่วงระยะเวลา 1 เดือนที่ผ่านมา

__________ น้อยกว่า 1 ครั้งต่อสัปดาห์

__________ 1 หรือ 2 ครั้งต่อสัปดาห์

__________ 3 ครั้งต่อสัปดาห์ขึ้นไป

1. ในช่วงระยะเวลา 1 เดือนที่ผ่านมา ท่านคิดว่าคุณภาพการนอนหลับโดยรวมของท่านเป็นอย่างไร

__________ ดีมาก

__________ ค่อนข้างดี

__________ ค่อนข้างแย่

__________ แย่มาก

1. ในช่วงระยะเวลา 1 เดือนที่ผ่านมา ท่านใช้ยาเพื่อช่วยในการนอนหลับ บ่อยเพียงใด (ไม่ว่าจะตามใบสั่ง

แพทย์ หรือ หาซื้อมาเอง)

__________ ไม่เคยเลยในช่วงระยะเวลา 1 เดือนที่ผ่านมา

__________ น้อยกว่า 1 ครั้งต่อสัปดาห์

__________ 1 หรือ 2 ครั้งต่อสัปดาห์

__________ 3 ครั้งต่อสัปดาห์ขึ้นไป

1. ในช่วงระยะเวลา 1 เดือนที่ผ่านมา ท่านมีปัญหาง่วงนอนหรือเผลอหลับ ขณะขับขี่ยานพาหนะ ขณะ

รับประทานอาหาร หรือขณะเข้าร่วมกิจกรรมทางสังคมต่างๆบ่อยเพียงใด

__________ ไม่เคยเลยในช่วงระยะเวลา 1 เดือนที่ผ่านมา

__________ น้อยกว่า 1 ครั้งต่อสัปดาห์

__________ 1 หรือ 2 ครั้งต่อสัปดาห์

__________ 3 ครั้งต่อสัปดาห์ขึ้นไป

1. ในช่วงระยะเวลา 1 เดือนที่ผ่านมา ท่านมีปัญหาเกี่ยวกับความกระตือรือร้นในการทำงานให้สำเร็จมาก

น้อยเพียงใด

__________ ไม่มีปัญหาเลยแม้แต่น้อย

__________ มีปัญหาเพียงเล็กน้อย

__________ ค่อนข้างที่จะเป็นปัญหา

__________ เป็นปัญหาอย่างมาก

1. ท่านมีคู่นอน เพื่อนร่วมห้องหรือผู้อาศัยอยู่ในบ้านหลังเดียวกันหรือไม่

__________ ไม่มีเลย

__________ มี แต่นอนคนละห้อง

__________ มี และนอนในห้องเดียวกัน แต่คนละเตียง

__________ มี และนอนเตียงเดียวกัน

หากท่านตอบว่ามี กรุณาสอบถามจากบุคคลข้างต้นว่า ในช่วงระยะเวลา 1 เดือนที่ผ่านมา ท่านได้เคยมี

อาการ ดังต่อไปนี้หรือไม่

- 1. กรนเสียงดัง

__________ ไม่เคยเลยในช่วงระยะเวลา 1 เดือนที่ผ่านมา

__________ น้อยกว่า 1 ครั้งต่อสัปดาห์

__________ 1 หรือ 2 ครั้งต่อสัปดาห์

__________ 3 ครั้งต่อสัปดาห์ขึ้นไป

- 1. มีช่วงหยุดหายใจเป็นระยะเวลานาน ขณะหลับ

__________ ไม่เคยเลยในช่วงระยะเวลา 1 เดือนที่ผ่านมา

__________ น้อยกว่า 1 ครั้งต่อสัปดาห์

__________ 1 หรือ 2 ครั้งต่อสัปดาห์

__________ 3 ครั้งต่อสัปดาห์ขึ้นไป

- 1. ขากระตุกขณะหลับ

__________ ไม่เคยเลยในช่วงระยะเวลา 1 เดือนที่ผ่านมา

__________ น้อยกว่า 1 ครั้งต่อสัปดาห์

__________ 1 หรือ 2 ครั้งต่อสัปดาห์

__________ 3 ครั้งต่อสัปดาห์ขึ้นไป

- 1. สับสนเป็นช่วงๆ ขณะหลับ

__________ ไม่เคยเลยในช่วงระยะเวลา 1 เดือนที่ผ่านมา

__________ น้อยกว่า 1 ครั้งต่อสัปดาห์

__________ 1 หรือ 2 ครั้งต่อสัปดาห์

__________ 3 ครั้งต่อสัปดาห์ขึ้นไป

- 1. อาการกระสับกระส่ายอื่นๆที่พบขณะหลับ ถ้ามี กรุณาระบุ

จากอาการในข้อ 10.5 ในช่วงระยะเวลา 1 เดือนที่ผ่านมา มีอาการบ่อยเพียงใด

__________ ไม่เคยเลยในช่วงระยะเวลา 1 เดือนที่ผ่านมา

__________ น้อยกว่า 1 ครั้งต่อสัปดาห์

__________ 1 หรือ 2 ครั้งต่อสัปดาห์

__________ 3 ครั้งต่อสัปดาห์ขึ้นไป
